# Cross-linked multifunctional binder in situ tuning solid electrolyte interface for silicon anodes in lithium ion batteries

**DOI:** 10.1038/s41598-023-45763-3

**Published:** 2023-10-29

**Authors:** Xiaofei Lou, Yuanyuan Zhang, Li Zhao, Teng Zhang, Hui Zhang

**Affiliations:** 1 College of Mechatronic Engineering, North Minzu University, Yinchuan, 750021 Ningxia China; 2 College of Pharmacy, Ningxia Medical University, Yinchuan, 750004 China; 3 State Key Laboratory of High-efficiency Utilization of Coal and Green Chemical Engineering, College of Chemistry and Chemical Engineering, Ningxia University, Yinchuan, 750021 Ningxia China

**Keywords:** Materials for energy and catalysis, Structural properties

## Abstract

Silicon is considered as the most promising anode material for high performance lithium-ion batteries due to its high theoretical specific capacity and low working potential. However, severe volume expansion problems existing during the process of (de)intercalation which seriously hinders its commercial progress. Binder can firmly adhere silicon and conductive agent to the current collector to maintain the integrity of the electrode structure, thereby effectively alleviating the silicon volume expansion and realizing lithium-ion batteries with high electrochemical performance. In this paper, citric acid (CA) and carboxymethyl cellulose (CMC) are adopted to construct a covalently crosslinked CA@CMC binder by an easy-to-scale-up esterification treatment. The Si@CA@CMC-1 electrode material shows an impressive initial coulombic efficiency (ICE) at 82.1% and after 510 cycles at 0.5 A/g, its specific capacity is still higher than commercial graphite. The excellent electrochemical performance of Si@CA@CMC-1 can be attributed to the ester bonds formed among CA@CMC binder and silicon particles. Importantly, by decoupling in situ EIS combining XPS at different cycles, it can be further proved that the CA@CMC binder can tune the component of SEI which provide a new-route to optimize the performance of silicon.

## Introduction

Silicon has long been considered to be one of the most promising anode materials for the next generation of lithium-ion batteries (LIBs)^1–4^. However, the inevitable volume expansion of silicon during (de)lithiation leads to structure crushing and the formation of unstable solid electrolyte interface (SEI), resulting in drastic capacity degradation^5–7^. Researchers in recent years have mainly focused on designing advanced structure to release volume expansion of silicon and constructing artificial SEI to stabilize cycling stability of silicon-based LIBs^8^. Critical role of binders is recognized to decrease irreversible capacity losses and promote the cycling life of batteries^9^. However, conventional polyvinylidene fluoride (PVDF) binder has induced van der waals’ force which are not strong enough for silicon electrode to endure its large volume expansions^10,11^.

An appropriate binder to achieve high performances of silicon electrode should be helpful to form strong interactions among active silicon materials for a stable long cycling life^12–16^. To improve adhesion between electrode components, many researchers have proposed a variety of functional one-component binders, including natural and synthetic binders^17,18^. One-component binders with functional groups can improve the adhesion to silicon particles by forming hydrogen bonds or covalent chemical bonds with the SiO_2_ layer on the surface of silicon and reduce the electrode crushing, thus improving the capacity retention to some extent^19–22^. Sodium carboxymethyl cellulose (CMC) is a natural binder which is composed of a large number of hydroxyl groups (–OH) and carboxymethyl groups (–OCH_2_COONa). Hochgatter et al.^23^ proved that the carboxylic acid group in CMC can react with –OH from silicon particles to form chemical bonds. The strong chemical interactions among CMC and silicon particles play a key role in maintaining long-cycle stability of the silicon electrode^24^. Zhang et al.^25^ has adopted phytic acid (PA) as a crosslinker to graft a covalently crosslinked binder CMC/PA. The abundant hydroxyl groups on the branched chain of PA can form hydrogen bonds with CMC and hydroxylated silicon nanoparticles. As a result, the Si-CMC/PA electrode shows a high reversible capacity of 2658.6 mAh/g after 70 cycles at a current density of 0.5 A/g and maintains a capacity of 1070.4 mAh/g after 450 cycles even at a high current of 4 A/g. It can be concluded that the formation of chemical bonds is one of the key factors to improve the cycling performance of silicon electrode and suitable small molecule crosslinked with CMC can further optimize stability.

Another challenge to optimize the performance of silicon electrode is to stabilize the formation of SEI during electrochemical process^26^. Commonly, SEI is composed of a soft porous organic layer contacting with electrolyte and a denser inorganic layer near the silicon electrode. The inorganic layer is made up of LiF, Li_2_O and Li_2_CO_3_ allowing Li^+^ transport which are postulated to be assembled in a mosaic-like pattern^27^. Porous organic layer outside with Li_2_EDC and ROLi (R depending on the solvent) are permeable to Li^+^ and electrolyte. Designing constitutionally stable SEI is an effective way to enhance ICE and prolong lifespan of silicon electrode. Adopting additives such as fluoroethylene carbonate (FEC), vinylene carbonate (VC), methylene ethylene carbonate (MEC) etc., into electrolyte is the traditional effective method to improve the cycling performance of silicon^28^. These additives give a rise to a SEI consisting of polycarbonate and inorganic salts through the surface reduction, which can be beneficial to further alleviate the reduction of solvent in the electrolyte. Another method is to introduce artificial SEI, improving the battery coulombic efficiency by pre-lithiation to compensate for lithium loss in lithium-ion batteries^29^. Though these methods are significant effective, complexity fabrication process and uncontrollable reaction will make a higher cost and lend additional uncertainty^30^.

In this paper, citric acid (CA) has been adopted as crosslinker to construct CA@CMC binder and different degrees of esterification are prepared by adjusting the concentration of carboxylic acid group. The Si@CA@CMC-X electrode is characterized to study structural morphology and the electrochemical test as well as decoupling interface component are carried out to clarify the adjustment mechanism of SEI induced by CA@CMC binder. This in situ cross-linked CA@CMC binder has improved performance for next-generation silicon-based LIBs and other energy storage devices.

## Experimental

### Materials

Citric acid (CA, Macklin), Carboxymethyl cellulose (CMC, Macklin), Carbon Black (CB, Macklin), Silicon (99.9%, D = 30–50 nm, Macklin).

### Electrode fabrication

A mass ratio of CA and CMC are added to deionized water and magnetically stirred for 30 min at room temperature to obtain a uniform aqueous solution of different proportions for CA@CMC-X, where X is the dosage of CA and CMC (Table S1). The electrode are m(silicon):m(carbon black):m(CA@CMC-X) = 8:1:1 (in mass ratio) and the areal density of the active material is ~ 0.4 mg/cm^2^. The above electrodes are cut into discs of 12 mm diameter and the final electrodes are assembled in a glove box (H_2_O < 0.01 ppm, O_2_ < 0.01 ppm) with lithium metal as the counter electrode and Celgard 2400 as the separator. The electrolyte adopted in this paper is 1 M LiPF_6_ (V(ethylene carbonate (EC))/V(dimethyl carbonate (DMC)) = 1:1).

### Structure and electrochemical characterization

#### Structure characterization

Diffuse Reflectance Infrared Fourier Transform Spectroscopy (DRIFTS, iS20, ThermoFisher); X-ray photoelectron spectroscopy (XPS, K-Alpha, Thermo Scientific); Scanning electron microscope (SEM, Gemini 300, ZEISS).

#### Electrochemical characterization

Galvanostatic charge–discharge (GCD) tests are performed on Neware CT-3008-S4 station in the range of 0.01–1.0 V at the current density of 0.5 A/g (0.1 A/g for the first 10 cycles). Electrochemical impedance spectroscopy (EIS) is measured in the frequency range from 100 mHz to 100 kHz at open circuit potential using Zennium XC electrochemical workstation. The parameters to calculate *D*_Li_^+^ are based on galvanostatic intermittent titration (GITT) tests (at 0.1 A/g with pulse time 20 min and relaxation time 30 min). To demonstrate the interface reaction mechanism of Si@CA@CMC electrode, in situ EIS at different potential are tested and the results are decoupled by distribution of relaxation time (DRT) tools provided by Ciucci’s group^31,32^.

## Results and discussion

**Figure 1:**
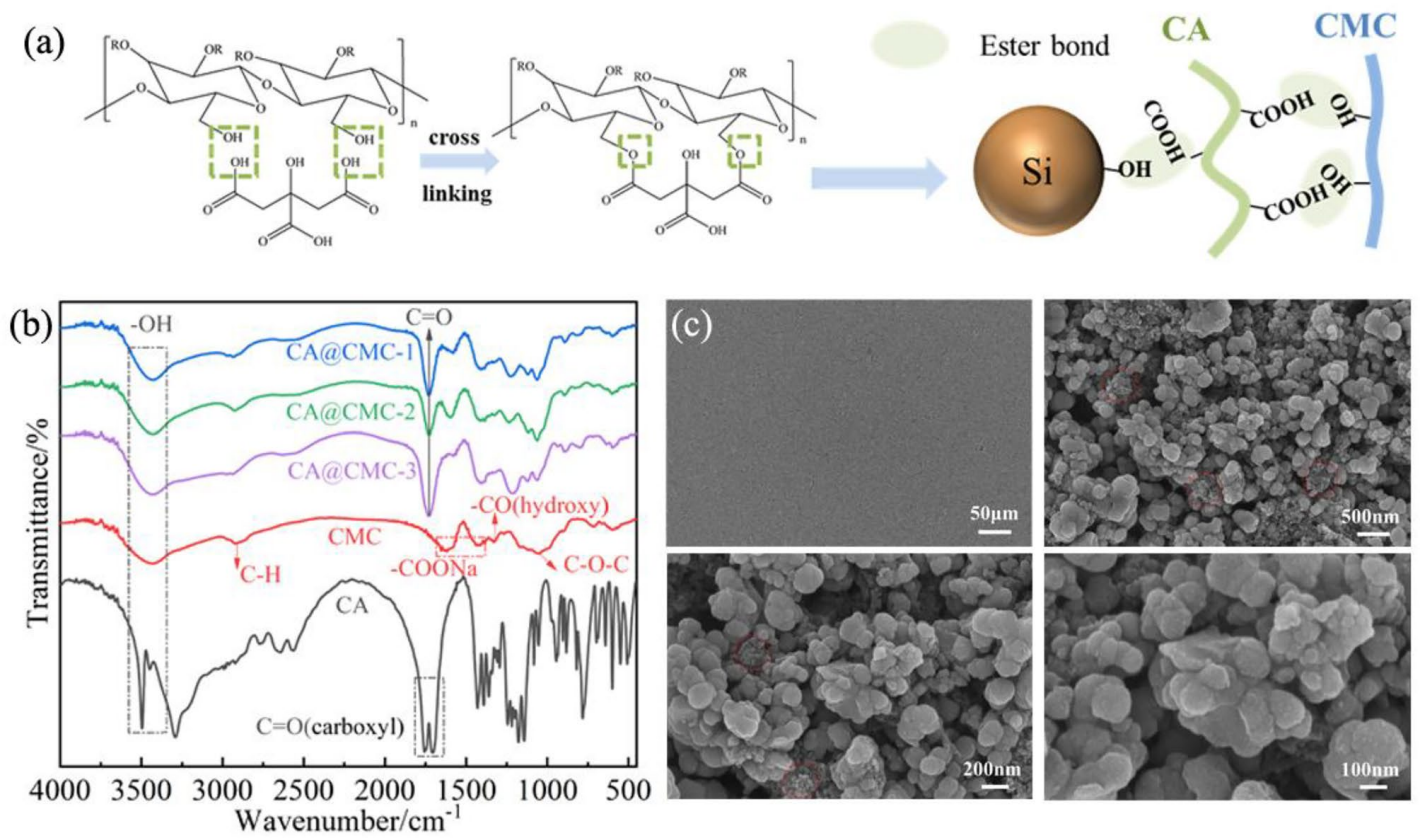
(**a**) The preparation schematic diagram of Si@CA@CMC electrode; (**b**) FTIR picture of CA, CMC and CA@CMC-X; (**c**) SEM images at different magnifications of Si@CA@CMC-1 electrode composite (fresh).

Carboxymethyl cellulose (CMC) is a natural binder composed of amply hydroxyl groups (–OH) and carboxymethyl groups (–OCH_2_COONa) while small molecule citrate acid (CA) contains in carboxyl groups (–COOH)^28,33,34^. The formation mechanism of Si@CA@CMC electrode is shown in Fig. 1a. The –COOH in CA is introduced into the rigid CMC main chain with the drying process at 80 ℃ which forms a cross-linked network containing covalent ester bonds generated by the chemical reaction of –OH in CMC and –COOH in CA. Meanwhile, some free –COOH groups from CA in the CA@CMC binder forms a covalent cross-linking network with the –OH of the silicon surface oxide layer. With these ester bonds among silicon particles and binders, dual-crosslinking network is successfully built. Covalent ester bonds are the strongest intermolecular interaction force which can withstand the large volume effect of silicon particles and ensure the integrity of the electrode structure during cycling^35^.

**Figure 2:**
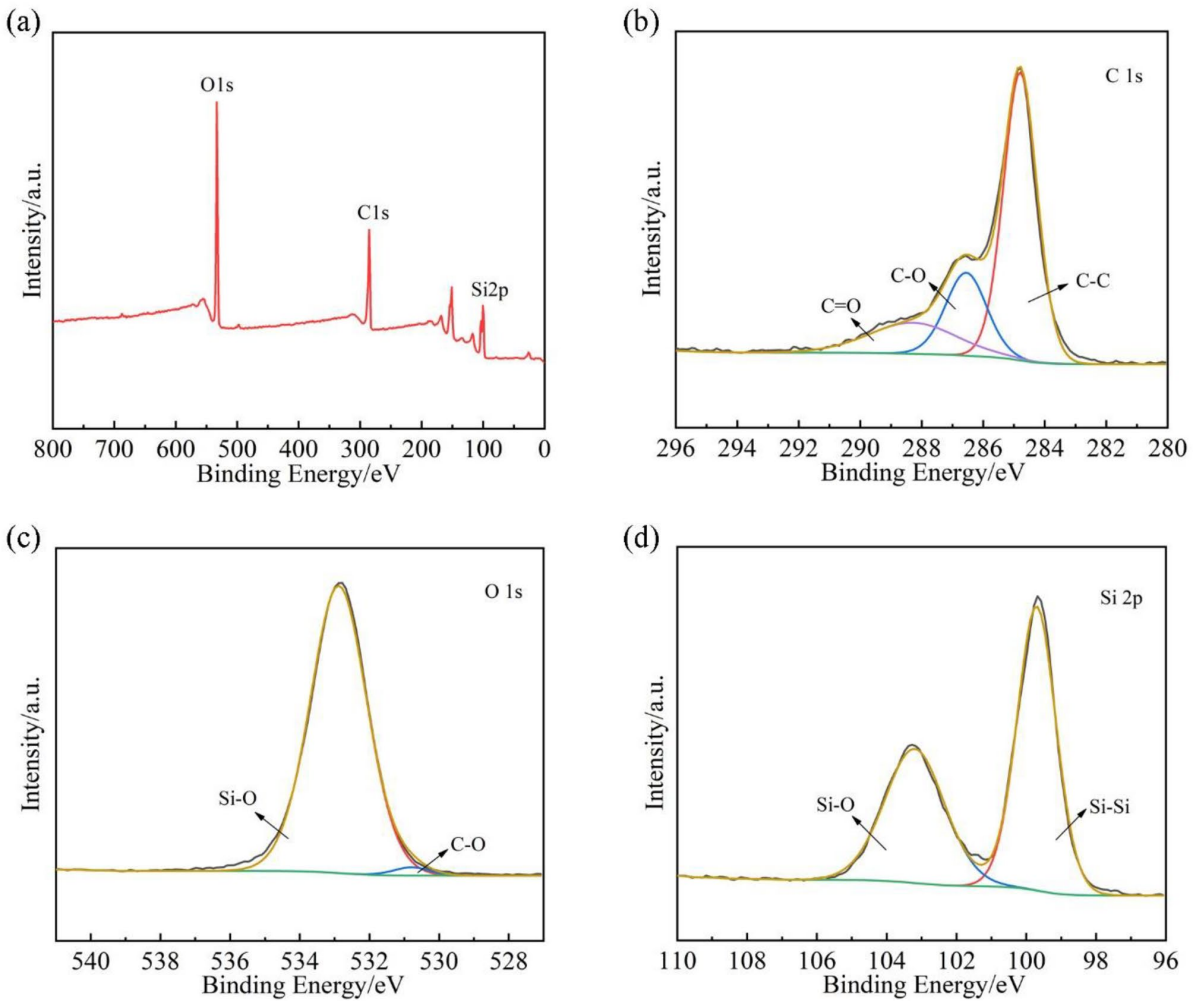
(**a**) Full spectrum of XPS of Si@CA@CMC-1 (fresh); (**b**) C 1s high-resolution spectrum; (**c**) O 1s high-resolution spectrum; (**d**) Si 2p high-resolution spectrum.

Figure 1b shows the Fourier transform infrared (FTIR) spectra of CA, CMC, different proportions of CA@CMC-X binder, silicon particles and Si@CA@CMC-1 composite. The narrow peaks around 3400–3500/cm are –OH vibration peaks from water molecules. Two peaks located at 1757/cm and 1703/cm correspond to the symmetric and asymmetric telescopic vibrations of C=O in the carboxylic acid group which demonstrates that CA contains multiple carboxylic acid groups. For CMC, a broad peak centered at 3429/cm is the –OH stretching vibration peak from water and the absorption peak at 2922/cm attributes to the stretching vibration of C-H. The absorption peaks around 1617/cm and 1422/cm are caused by -COONa (asymmetry) and –COONa (symmetry) whilst the peak at 1325/cm is the –CO from hydroxyl group, proving that CMC contains a certain amount of hydroxyl group. The infrared spectra of different proportions of CA@CMC-X show similar characteristic peaks around 3420–3435/cm, representing –OH stretching vibration. And the peak of the ester carbonyl group C=O is shown at 1726–1734/cm, indicating that esterification reaction has been generated among the carboxylic acid groups in CA and the hydroxyl groups in CMC^33^. SEM method is used to characterize the surface topography of the Si@CA@CMC-1 electrode material before cycling and the results can be shown in Fig. 1c. The particle size distribution of the silicon nanoparticles is relatively uniform in the range of 30–50 nm, which matches well with pure silicon. In addition, spherical silicon nanoparticles are tightly connected with each other which will generate some void space to relieve the volume stress generated by volume effect. The full spectrum of XPS of Si@CA@CMC-1 is presented in Fig. 2a and sharp peaks can be found at 285.08 eV, 533.08 eV and 100.08 eV, indicating that the material contains C, O and Si elements. Figure 2b shows the high-resolution spectrum of C 1s which is decomposed into three peaks centered at 284.8 eV, 286.58 eV and 288.48 eV, corresponding to C–C, C–O, C=O bonds, respectively. O 1s spectra (Fig. 2c) proposes two peaks at 530.68 eV and 532.88 eV representing C–O and Si–O bonds. Two peaks for Si 2p spectra at Fig. 2d can be decomposed two peaks at 99.68 eV and 103.18 eV, standing for Si–Si and Si–O bonds^36^. The presence of Si–O bonds indicates the presence of SiO_x_ layer on the surface of the silicon particles, which is caused by the slight oxidation of silicon exposed to air. The chemical bonds of Si@CA@CMC-X are proposed in Tables S2 and S3. The content of C=O groups is 17.22%, 16.13%, 15.45% for Si@CA@CMC-1/2/3, respectively, which proves that Si@CA@CMC-1 has the highest esterification degree and the dosage of CA is the key to tune the crosslinked process.

**Figure 3:**
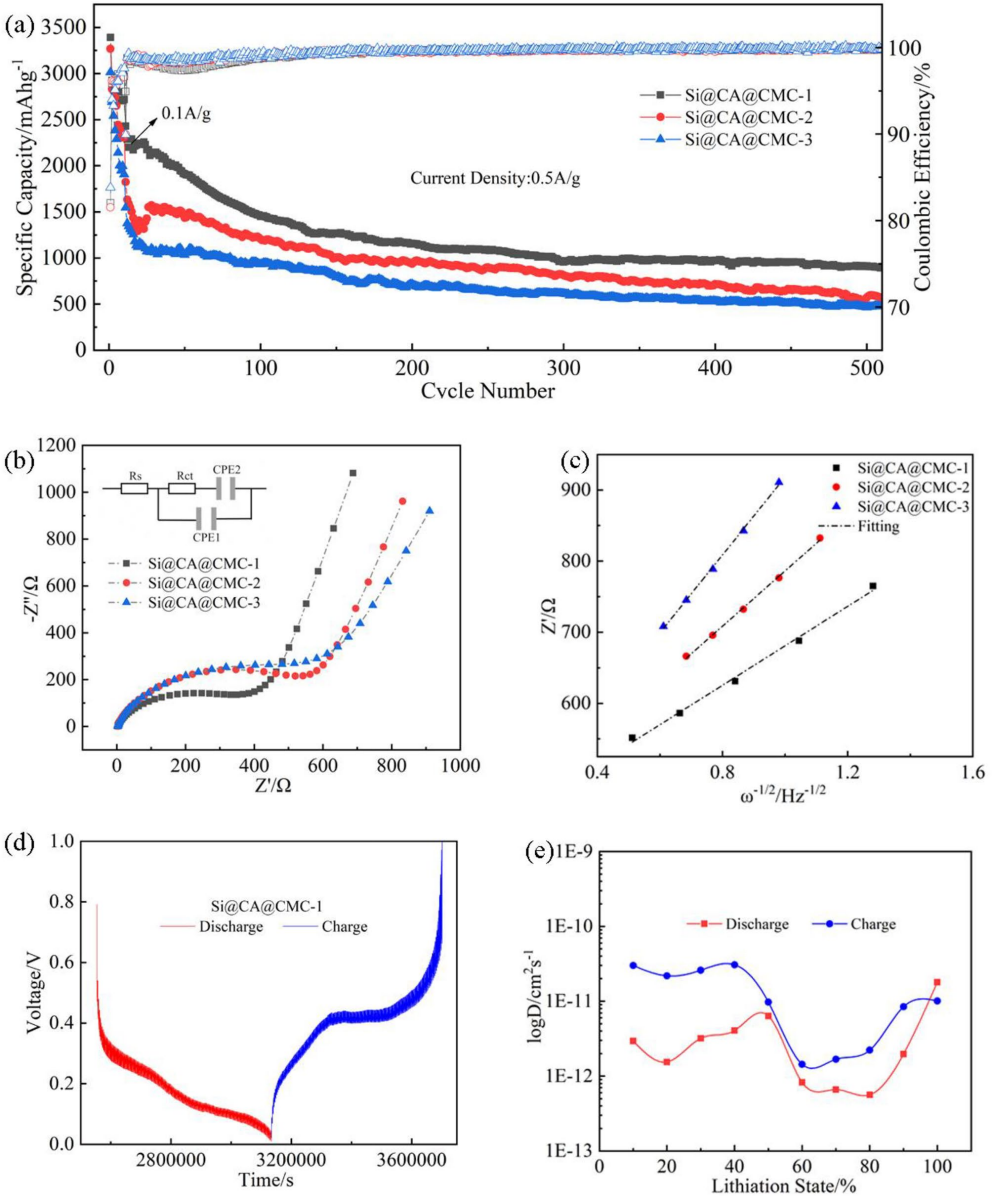
(**a**) Cycling performance of Si@CA@CMC-1/2/3 electrodes at 0.5A/g; (**b**) Nyquist plots of Si@CA@CMC-1, Si@CA@CMC-2 and Si@CA@CMC-3 at open circuit potential; (**c**) the relationship between Z′ and ω^−1/2^ at a very low frequency region; (**d**) GITT curves for Si@CA@CMC-1; (**e**) *D*_Li_^+^ during the discharge and charge process at different (de)lithiation state of Si@CA@CMC-1.

Galvanostatic charge–discharge test of Si@CA@CMC-X electrode materials are shown in Fig. 3a. 0.1 A/g current density has been adopted during the first ten cycles for activation. The first discharge specific capacity of Si@CA@CMC-1, Si@CA@CMC-2 and Si@CA@CMC-3 electrodes is 3390.57 mAh/g, 3268.12 mAh/g, 3015.97 mAh/g, respectively, whilst the initial coulombic efficiency (ICE) is 82.1%, 81.5%, 83.2%. After nearly 500 cycles, the capacity retention ratios are 71.63%, 55.78% and 51.3%, respectively, calculated based on the first circle. It can be found out that the capacity retention of Si@CA@CMC-1 is much higher than other two electrode, certifying the fixation effectives of CA@CMC-1 binder is helpful to release volume expansion of silicon during electrochemical process. In addition, the coulombic efficiency of Si@CA@CMC-1 is up to 99.91% while it’s 99.89% and 99.82% for Si@CA@CMC-2 and Si@CA@CMC-3 after hundred cycles, proving the suitable binder structure designing can further be useful for enhancing coulombic efficiencies. To demonstrate the reaction kinetics of Si@CA@CMC-X electrodes, the differential capacitance curves (dQ/dV) are plotted as shown in Fig. S4d–f. No further reduction peak around 0.8 V in the negative sweep exists, indicating barely repeating growth of SEI, which further explains the high first ICE of the electrodes.

EIS tests have been carried out to investigate the effect of the electrode materials and the diffusion of Li^+^ (*D*_Li_^+^) has also been calculated in Fig. 3b, c. The Nyquist plots for Si@CA@CMC-X are similar which consists of a semi-circle and a straight line. The charge transfer resistances (*R*_ct_) are 400Ω, 575Ω and 579Ω, respectively, which illustrates the better conductance for Si@CA@CMC-1. To intensively study the kinetic process of Si@CA@CMC-X electrodes, the diffusion rate of Li^+^ is calculated according to the equation below^37^:

$$ \begin{aligned} D_{{{\text{Li}}^{ + } }} & = \frac{{R^{2} T^{2} }}{{2A^{2} n^{4} F^{4} C^{4} \sigma^{2} }} \\ Z & = {\text{R}}_{e} + R_{ct} + \sigma \varpi^{ - 1/2} \\ \end{aligned} $$

where R is the gas constant, T refers to the absolute temperature, A is the surface area of the electrode, n represents for the number of electrons per molecule during the electrode reaction, F is Faraday’s constant, C stands for the concentration of Li^+^ in the electrode, ω is the angular frequency, σ is the Warburg factor, which is the slope of Z' in this research and fitting linear with ω^−1/2^. The calculated *D*_Li_^+^ is 5.19 × 10^–16^ cm^2^/s, 2.64 × 10^–16^ cm^2^/s, 1.33 × 10^–16^ cm^2^/s. *D*_Li_^+^ which has proven the excellent lithium-ion diffusion properties for Si@CA@CMC-1.

Galvanostatic intermittent titration technique (GITT) method is another effective method to calculated *D*_Li_^+^. The calculation results are shown in Fig. 3d, e and Tables S5–S7. The adopted equation is as below^2^:

$$ D_{{{\text{Li}}^{ + } }} { = }\frac{{4{\text{L}}^{2} }}{\pi \tau }\left( {\frac{{\Delta {\text{E}}_{s} }}{{\Delta {\text{E}}_{t} }}} \right)^{2} $$

where *D* is the diffusion coefficient of lithium ion (cm^2^/s), L represents for the Li^+^ diffusion length (cm) which equals to the thickness of electrode, τ is the relaxation time (s), Δ*E*_s_ stands for the steady-state potential change (V) by the current pulse, Δ*E*_t_ refers to the potential change (V) during the constant current pulse after subtracting the IR drop. All the lithiation state *vs D*_Li_^+^ curves show a “W” type for both alloying and dealloying matching well with previous reports^38^. Detailly, during the lithiation process of Si@CA@CMC-X electrode, the *D*_Li_^+^ is distributed in the range of 3.14 × 10^–12^ to 2.24 × 10^–11^ cm^2^/s, 5.64 × 10^–13^ to 1.79 × 10^–11^ cm^2^/s and 1.44 × 10^–12^  to  3.07 × 10^–11^ cm^2^/s. Obviously, the Si@CA@CMC-1 electrode has higher *D*_Li_^+^ than other electrode which is in consistent with the results of EIS, testifying the fastest in ion transferring of SI@CA@CMC-1.

**Figure 4:**
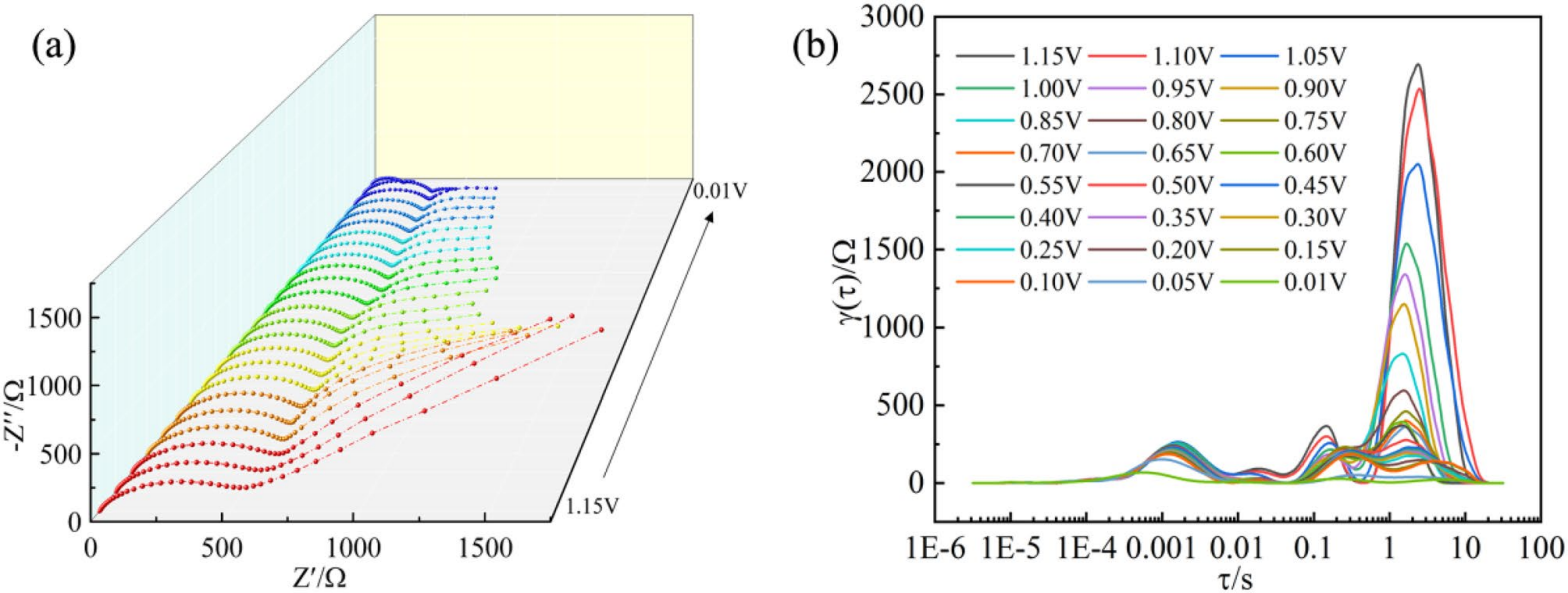
(**a**, **b**) In situ EIS Nyquist plots and the distribution of relaxation time (DRT) related function γ(τ) at different voltage in the first discharge process of Si@CA@CMC-1.

In situ EIS tests for the first cycles are performed to comprehensively demonstrate the electrochemical process and the kinetic information are analyzed by DRT (Distribution of Relaxation Time) tools provided by Ciucci’s group (Figs. 4 and S6)^31^. The high frequency region in the τ < 0.003 s range is related to the formation of solid-electrolyte interfaces (SEIs) and the peak value of the Si@CA@CMC-1 electrode in the high-frequency region decreases while the peak position is shifted to the left, indicating the CA@CMC-1 binder is more conducive to forming a stable passivation layer on the surface of silicon. The intermediate frequency region in the range of 0.003 s < τ < 1 s is caused by the charge transfer process at the interface between the SEI and the active material of the electrode. It can be found that the peaks intensities at 0.003 s < τ < 1 s of Si@CA@CMC-1 electrode from 1.15 to 0.85 V has been continuously decreased and shifted to the right, which manifests the formation of SEI. The region in the range of 1 s < τ < 10 s is related to the intercalation of silicon and the peak value decrease sharply for the formation of Li-Si alloy and the peaks intensities has become lowered, proving the alloying process of silicon.

**Figure 5:**
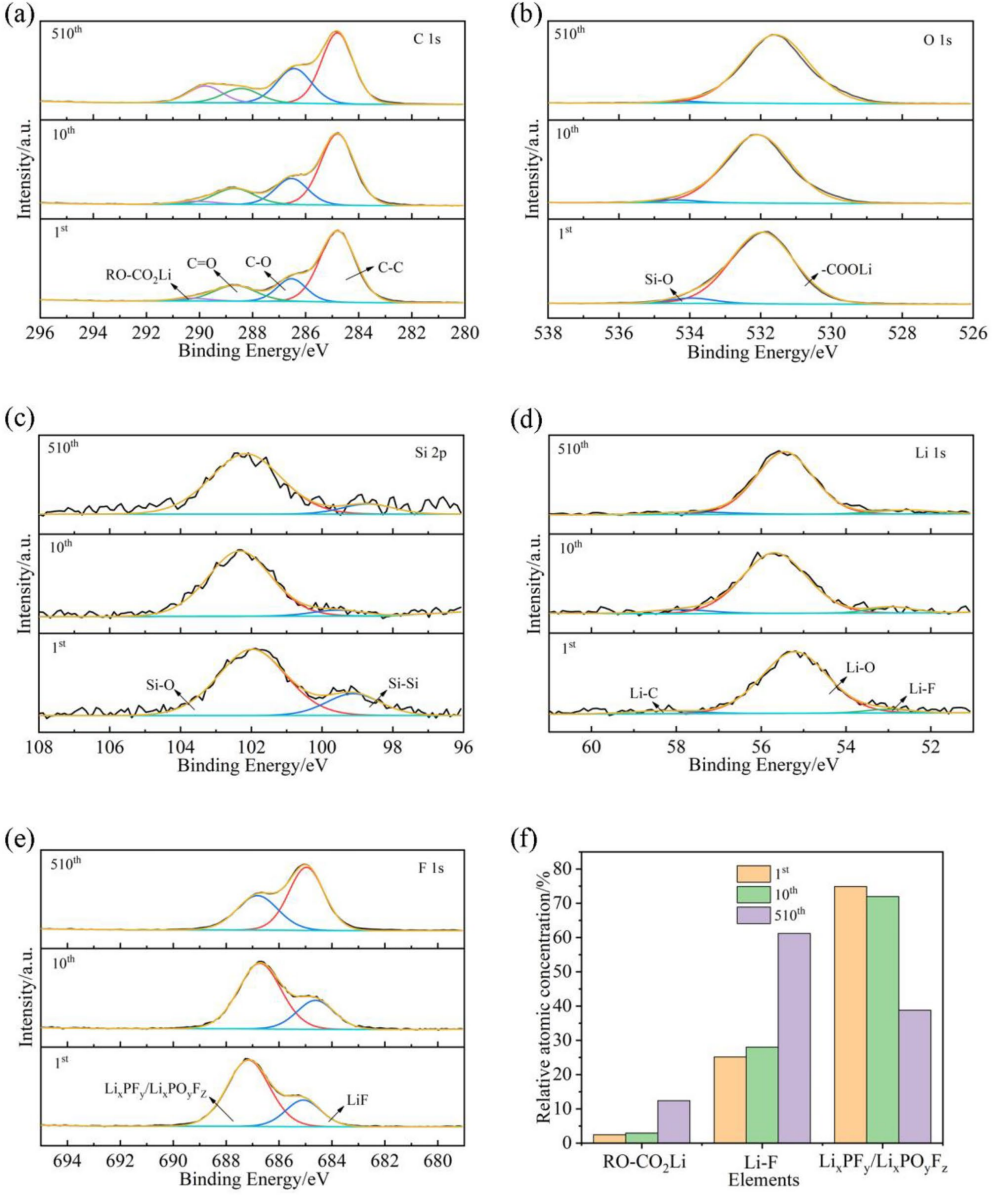
(**a**) C 1s fine spectrum; (**b**) O 1s fine spectrum; (**c**) Si 2p fine spectrum; (**d**) Li 1s fine spectrum; (**e**) F 1s fine spectrum and (**f**) Chemical bonds contents histogram calculated from XPS of Si@CA@CMC-1 after 1,10, 510 cycles (disassembled at charge state (1 V)).

**Figure 6:**
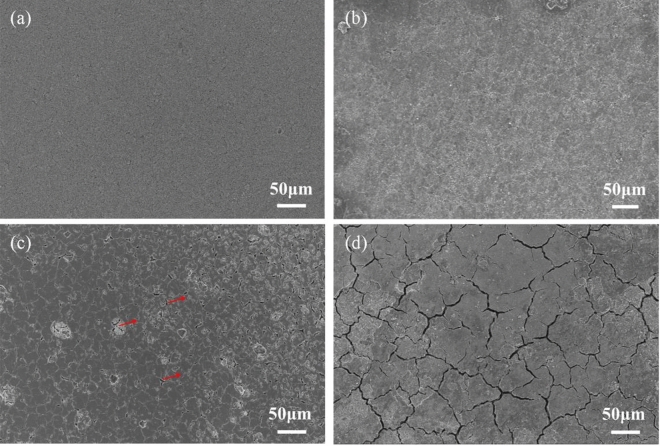
SEM images of Si@CA@CMC-1 electrode material without further treatment: (**a**) fresh; (**b**) 1st cycle; (**c**) 10th cycles; (**d**) 510th cycles (at charge state (1 V)).

The postmortem of Si@CA@CMC-1 after different cycles has been obtained at lithiation state and high-resolution XPS spectra of C 1s, O 1s, F 1s, Si 2p and Li 1s (Fig. 5) are performed to figure out the transformation of interface chemical state during electrochemical process. Four peaks at 284.80 eV, 286.58 eV, 288.68 eV, 290.28 eV belong to C–C, C–O, C=O, RO-CO_2_Li, and for O 1s spectrum including two peaks at 531.88 eV and 533.98 eV are characteristic peaks of –COOLi and Si–O bonds, whilst peaks at 99.08 eV and 101.98 eV can be assigned to Si–O and Si–Si chemical bonds, respectively^39^. F element are explored for the decomposition of LiPF_6_ and two peaks at 684.98 eV and 686.78 eV are observed in the F1s spectrum, representing Li–F and Li_x_PF_y_/Li_x_PO_y_F_Z_^40^. In the Li 1s spectrum, peak is deconvoluted into three typical peaks corresponding to Li–F bond at 52.98 eV, Li–O bond at 55.48 eV and Li–C bond at 58.18 eV^25^. SEI is composed of organic layer and inorganic layer and the content of RO-CO_2_Li has increased from 2.24 to 12.37% with the lithiation process continuing, in consistent with the increase in the content of –COOLi in O1s. Meanwhile, the content of Li_x_PF_y_/Li_x_PO_y_F_Z_-LiF has increased indicating the CA@CMC-1 binder effectively regulates the organic and inorganic contents in the SEI layer^41^. Furthermore, LiF has been regarded as an important inorganic component for SEI due to its compact structure and benign mechanical stability to prevent the decomposition of electrolyte. As Table S9 shows, the content of LiF for Si@CA@CMC-1 has increased from 25.12 to 61.2%, indicating a dense layer SEI layer is formed on the electrode surface. In addition, Li_x_PF_y_/Li_x_PO_y_F_Z_ are decomposition of electrolytes and the decreasing contents indicate that the CA@CMC-1 binder inhibits the reduction of electrolytes to form stable SEI which promotes the cycling stability of silicon electrodes. In brief, the role of CA@CMC-1 binder has play dual functions role which is the fixation effective of silicon and beneficial to adjust the component of SEI to prolong cycle life and improve the ICE of silicon electrode. Batteries at different cycles are disassembled (charged to 1 V) and characterized by SEM in Fig. 6 to attain the morphological of the Si@CA@CMC-1 electrode. With the electrochemical process going, cracks are generated but still fixed on the copper foil which prove the CA@CMC-1 binder can be used to withstand high stress to avoid pulverization and ensure the stability of the electrode and electrical connection.

The carboxylic acid group –COOH in CA will cross-link and polymerize with the hydroxyl group –OH in CMC through esterification reaction to form a covalent cross-linking binder CA@CMC. Meanwhile, the unreacted –COOH in the CA@CMC binder would provide a multidimensional bonding point and successfully construct a covalent double-crosslinking network structure of the Si@CA@CMC composite electrode material. By adjusting the ratio of CA to CMC, the concentration of –COOH in the binder can be changed to form electrode materials with different degrees of esterification with silicon. Thus, the Si@CA@CMC-1 electrode has superior electrochemical performance with high first discharge specific capacity (3390.57/mAhg) and first Coulombic efficiency (82.03%) at a current density of 0.1/Ag. Moreover, the average decay rate per cycle was only 0.02% at the 300th–500th cycles at a current density of 0.5/Ag with good cycling stability. This excellent performance of Si@CA@CMC-1 is attributed to the abundance of –COOH sites in the CA@CMC-1 binder, which is helpful to improve the interfacial interactions between electrode components and increase the degree of esterification. The double crosslinked network electrode structure with the highest degree of esterification provides a more stable three-dimensional framework for the silicon nanoparticles, which helps the silicon to withstand higher stresses to avoid material pulverization, whilst ensures the structural integrity of the electrode sheet and the stability of the conductive network. What’s more, the reason for the high first efficiency of Si@CA@CMC-1 is that the addition of the binder not only enhances the structural stability of the electrode, but also regulates the interface of the silicon electrode as a functional component. Importantly, the CA@CMC-1 binder effectively regulates the SEI composition as well as the relative content, and the inorganic component LiF increases from 25.12 to 61.2% with the continuous cycling, which is conducive to the improvement of the toughness and stability of the SEI layer and the prevention of the silicon from continuing to be exposed to the electrolyte. The large reduction of the electrolyte decomposition products Li_x_PF_y_/Li_x_PO_y_F_Z_ indicates that the binder inhibits the electrolyte decomposition, which is conducive to the formation of a thinner and more stable SEI on the silicon surface, and enhances the cycling performance of the electrode.

## Conclusion

In this paper, citrate acid (CA) modified linear carboxymethylcellulose CMC is constructed to form aqueous CA@CMC binder. A high initial coulombic efficiency of 82.1% has been achieved by Si@CA@CMC-1 electrode and after 510 cycles at 0.5 A/g, the specific capacity of silicon is still higher than commercial graphite, showing a good cycle stability and reversibility. The comparable high performance can be attributed to the marvelous east-to-scale-up designing. Firstly, a stable conductive network is built by CA@CMC binder which is proposed by eater bonds generated by multiple –COOH from CA which works as a bridge to connect silicon and CMC. In addition, the ratio of inorganic LiF and organic Li_x_PF_y_/Li_x_PO_y_F_Z_ has been tuned, decreasing the decomposed of electrolyte and enhancing the stability of SEI to the higher ICE and long cycle life. This in situ adjustment of SEI method by constructing multi-functional binder provide a new strategy to optimize the performance of silicon.

### Supplementary Information

Supplementary Information.

## Data Availability

The datasets used and/or analyzed during the current study available from the corresponding author on reasonable request.
