# Supplementary material for: Cross-linked multifunctional binder in situ tuning solid electrolyte interface for silicon anodes in lithium ion batteries
Source: Sci Rep. 2023 Oct 29;13:18560. doi: 10.1038/s41598-023-45763-3 (PMC10613629; doi:10.1038/s41598-023-45763-3)
Supplement: Supplementary file 1 — Supplementary Information. [file 41598_2023_45763_MOESM1_ESM.docx]

**Cross-linked multifunctional binder *in situ* tuning solid electrolyte interface for silicon anodes in lithium ion batteries**

Xiaofei Lou^1*^, YuanYuan Zhang^2*^, Li Zhao^1^, Teng Zhang^1^, Hui Zhang^3^

^1^ College of Mechatronic Engineering, North Minzu University, Yinchuan 750021, Ningxia, China

^2^ College of Pharmacy, Ningxia Medical University, Yinchuan, 750004, China

^3^ State Key Laboratory of High-efficiency Utilization of Coal and Green Chemical Engineering, College of Chemistry and Chemical Engineering, Ningxia University, Yinchuan 750021, Ningxia, China

*E-mail: [feixiaolou@126.com](mailto:feixiaolou@126.com); smnxmu@126.com

**Table S1** Content of original material in CA@CMC-X binder

|  | CA@CMC-1 | CA@CMC-2 | CA@CMC-3 |
| --- | --- | --- | --- |
| CA | 5 mg | 3.3 mg | 6.7 mg |
| CMC | 5 mg | 6.7 mg | 3.3 mg |





**Figure S1** FTIR picture of pure Si, CA@CMC-1 and Si@CA@CMC-1


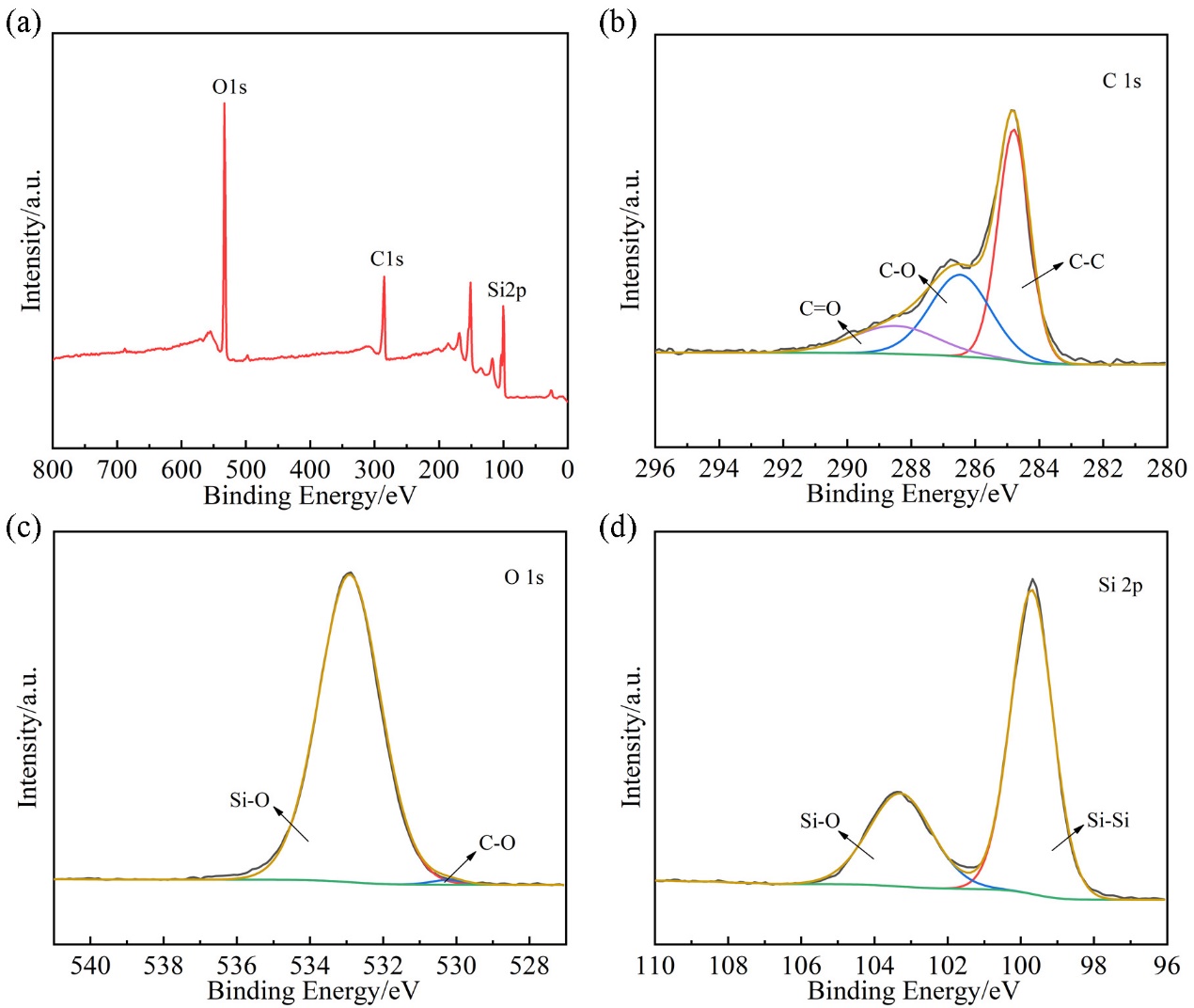


**Figure S2** (a) Full spectrum of XPS of Si@CA@CMC-2 (fresh); (b) C 1s fine spectrum; (c) O 1s fine spectrum; (d) Si 2p fine spectrum


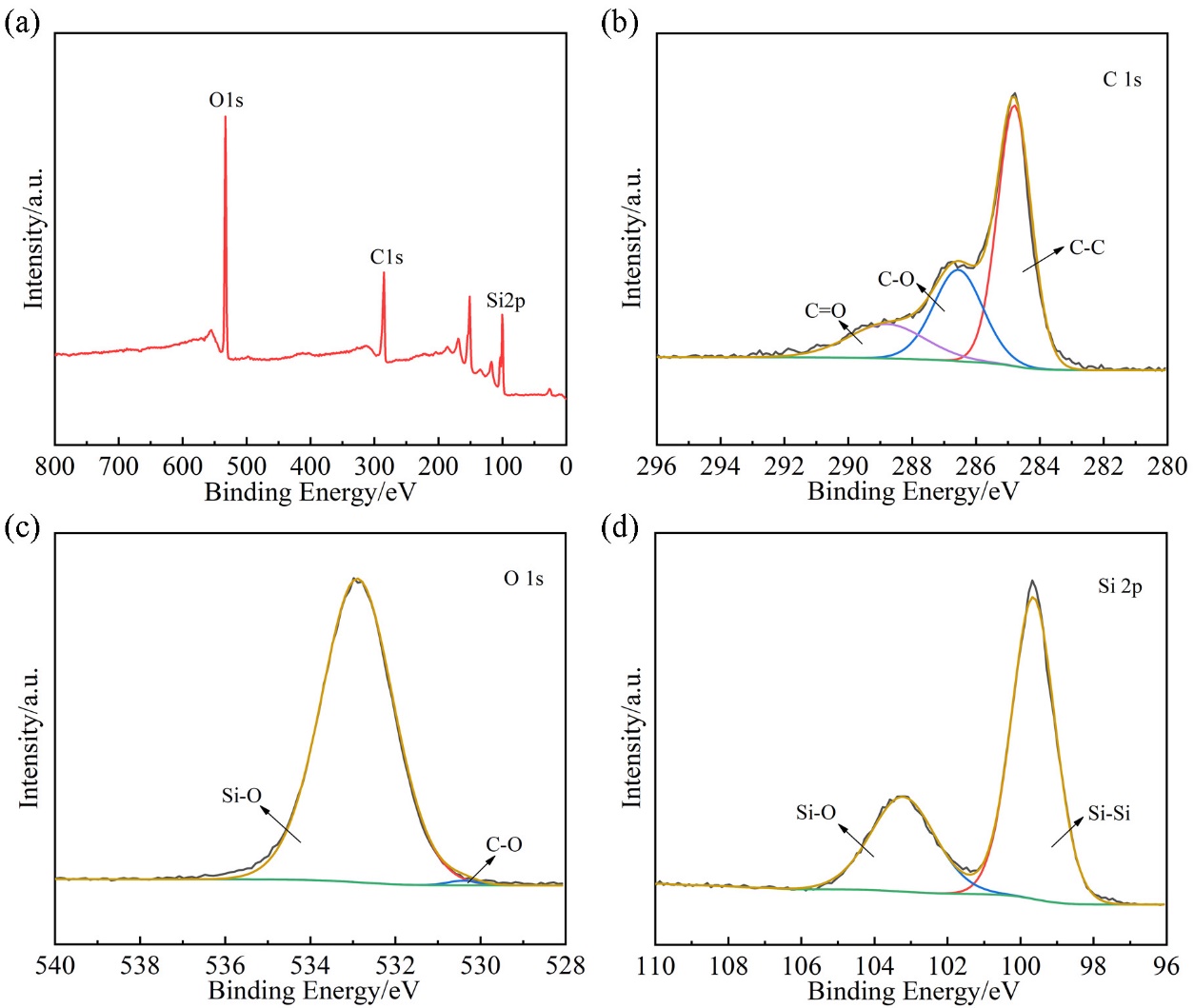


Figure **S3** (a) Full spectrum of XPS of Si@CA@CMC-3 (fresh); (b) C 1s fine spectrum; (c) O 1s fine spectrum; (d) Si 2p fine spectrum

**Table S2** Statistics of the atomic contents in XPS of Si@CA@CMC-1/2/3(fresh)

|  | C 1s | O 1s | Si 2p |
| --- | --- | --- | --- |
| Si@CA@CMC-1 | 29.24 | 52.92 | 17.84 |
| Si@CA@CMC-2 | 22.47 | 54.38 | 23.15 |
| Si@CA@CMC-3 | 25.36 | 53.41 | 21.23 |

**Table S3** Statistics of the chemical bond contents in XPS of Si@CA@CMC-1/2/3(fresh)

|  | | Si@CA@CMC-1 | Si@CA@CMC-2 | Si@CA@CMC-3 |
| --- | --- | --- | --- | --- |
| C 1s | C-C | 61.27 | 50.27 | 55.80 |
|  | C-O | 21.51 | 33.60 | 28.75 |
|  | C=O | 17.22 | 16.13 | 15.45 |
| O 1s | Si-O | 98.2 | 99.15 | 99.35 |
|  | C-O | 1.8 | 0.85 | 0.65 |
| Si 2p | Si-O | 42.26 | 31.57 | 31.86 |
|  | Si-Si | 57.74 | 68.43 | 68.14 |


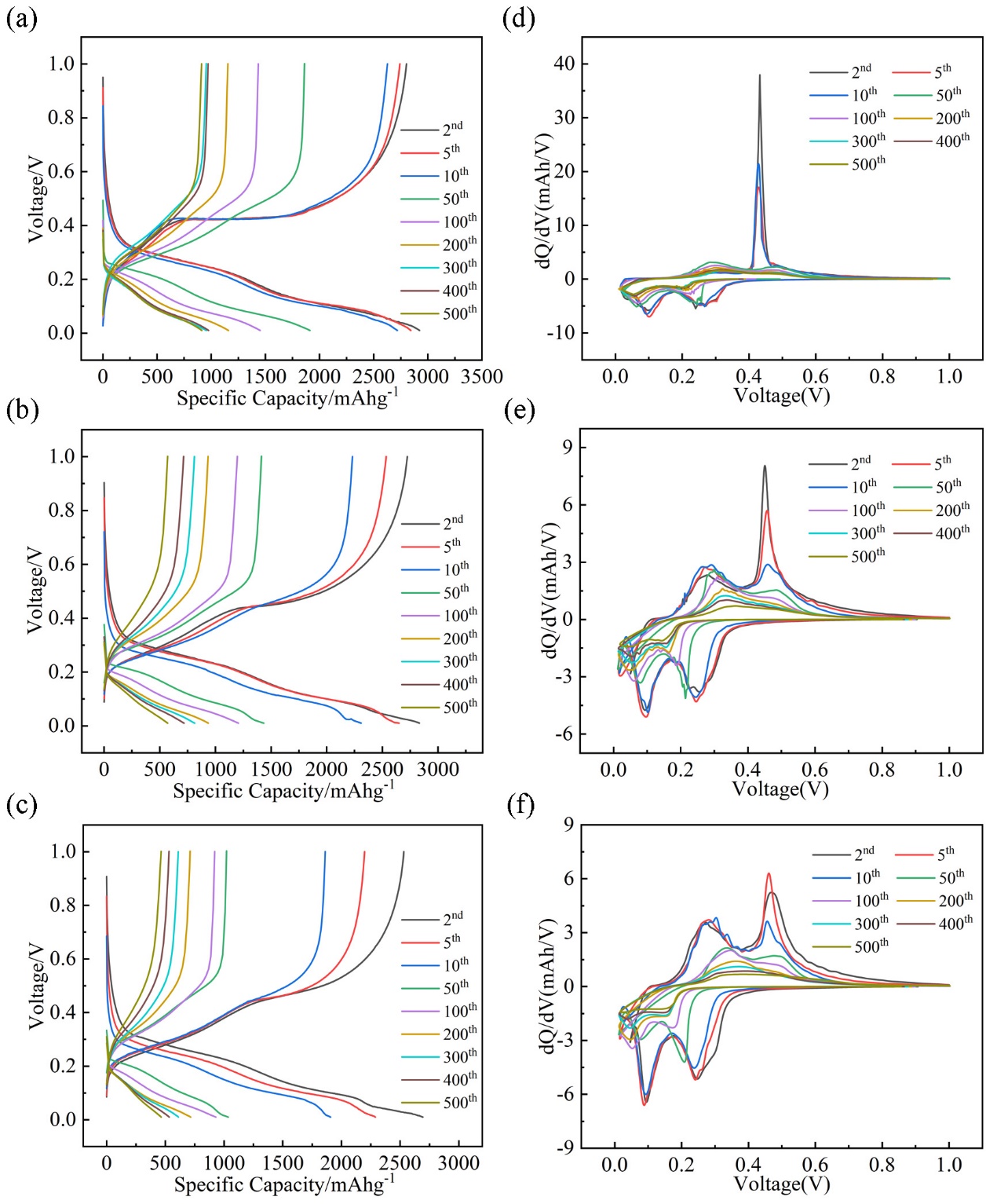


**Figure S4** Galvanostatic charge and discharge profiles and dQ/dV curves of (a)-(d) Si@CA@CMC-1；(b)-(e) Si@CA@CMC-2；(c)-(f) Si@CA@CMC-3 at different cycles

**Table S4** Each component value in EIS equivalent circuit and *D*_Li_^+^ statistics

|  | R_s_/Ω | R_ct_/Ω | CPE_1_/μF | CPE_2_/mF | σ/Ω/s^-1/2^ | D_Li+_/cm^2^s^-1^ |
| --- | --- | --- | --- | --- | --- | --- |
| Si@CA@CMC-1 | 2.46 | 400 | 391 | 13.3 | 277.33 | 5.19×10^-16^ |
| Si@CA@CMC-2 | 3.41 | 575 | 228 | 7.50 | 388.54 | 2.64×10^-16^ |
| Si@CA@CMC-3 | 2.45 | 579 | 207 | 8.42 | 547.04 | 1.33×10^-16^ |


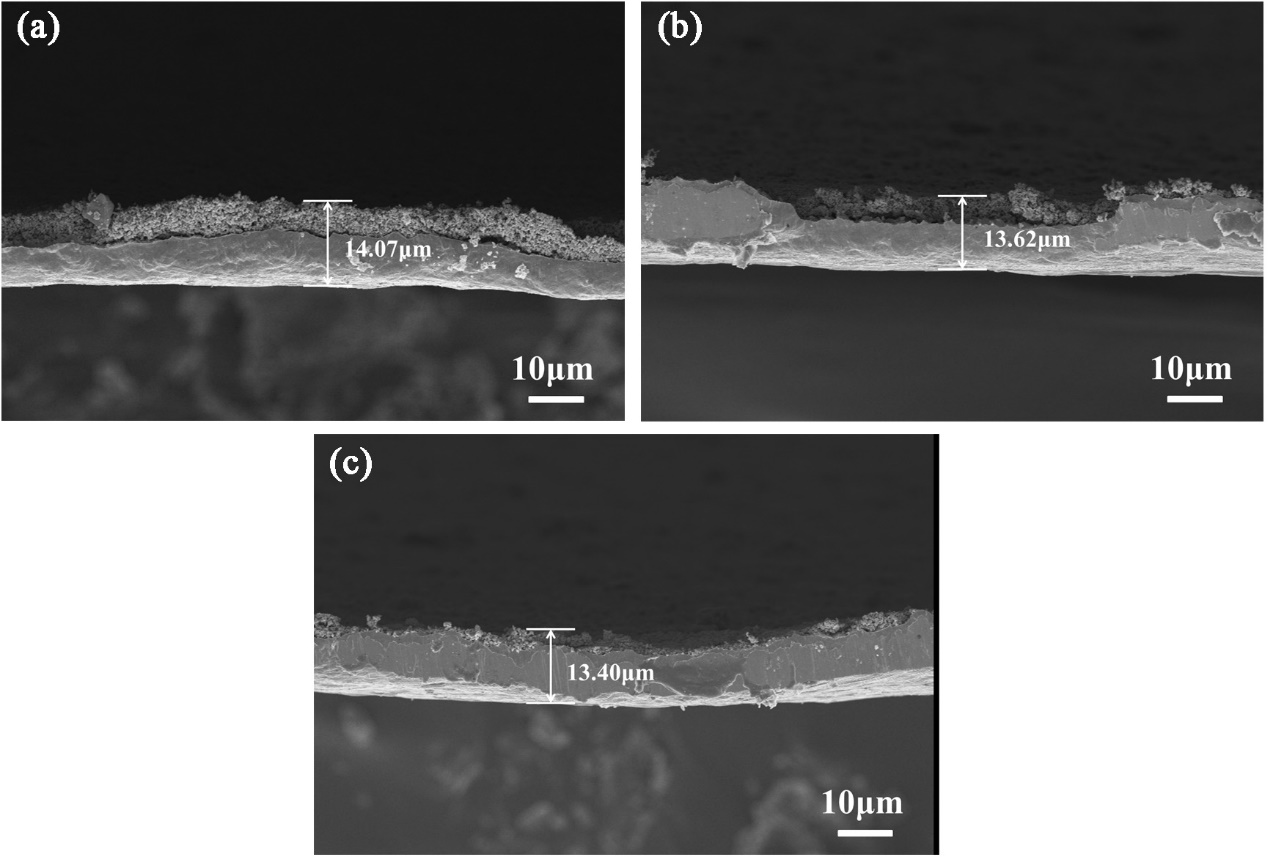


**Figure S5** Cross-sectional SEM images of (a) Si@CA@CMC-1; (b) Si@CA@CMC-2; (c) Si@CA@CMC-3 electrode

**Table S5** GITT data for Si@CA@CMC-1

| Lithiation | Constant | L/cm | π | τ | ΔE_t_/V | ΔE_s_/V | *D*_Li+_/cm^2^s^-1^ | |
| --- | --- | --- | --- | --- | --- | --- | --- | --- |
| 10% | 4 | 0.00141 | 3.14 | 1800 | 0.0626 | 0.0065 | 1.52×10^-11^ | |
| 20% | 4 | 0.00141 | 3.14 | 1800 | 0.0531 | 0.005 | 1.25×10^-11^ | |
| 30% | 4 | 0.00141 | 3.14 | 1800 | 0.0515 | 0.0065 | 2.24×10^-11^ | |
| 40% | 4 | 0.00141 | 3.14 | 1800 | 0.0468 | 0.0059 | 2.24×10^-11^ | |
| 50% | 4 | 0.00141 | 3.14 | 1800 | 0.0415 | 0.0034 | 9.44×10^-12^ | |
| 60% | 4 | 0.00141 | 3.14 | 1800 | 0.0381 | 0.0018 | 3.14×10^-12^ | |
| 70% | 4 | 0.00141 | 3.14 | 1800 | 0.0372 | 0.0018 | 3.29×10^-12^ | |
| 80% | 4 | 0.00141 | 3.14 | 1800 | 0.0391 | 0.0021 | 4.06×10^-12^ | |
| 90% | 4 | 0.00141 | 3.14 | 1800 | 0.0462 | 0.0037 | 9.02×10^-12^ | |
| 100% | 4 | 0.00141 | 3.14 | 1800 | 0.053 | 0.0038 | 7.23×10^-12^ | |
| Delithiation | Constant | L/cm | π | τ | ΔE_t_/V | ΔE_s_/V | *D*_Li+_/cm^2^s^-1^ | |
| 10% | 4 | 0.00141 | 3.14 | 1800 | 0.0319 | 0.0037 | 1.89×10^-11^ | |
| 20% | 4 | 0.00141 | 3.14 | 1800 | 0.0335 | 0.0031 | 1.20×10^-11^ | |
| 30% | 4 | 0.00141 | 3.14 | 1800 | 0.0366 | 0.0043 | 1.94×10^-11^ | |
| 40% | 4 | 0.00141 | 3.14 | 1800 | 0.0388 | 0.0049 | 2.24×10^-11^ | |
| 50% | 4 | 0.00141 | 3.14 | 1800 | 0.0385 | 0.0015 | 2.14×10^-12^ | |
| 60% | 4 | 0.00141 | 3.14 | 1800 | 0.0347 | 0.0012 | 1.68×10^-12^ | |
| 70% | 4 | 0.00141 | 3.14 | 1800 | 0.0413 | 0.0022 | 3.99×10^-12^ | |
| 80% | 4 | 0.00141 | 3.14 | 1800 | 0.0527 | 0.0017 | 1.46×10^-12^ | |
| 90% | 4 | 0.00141 | 3.14 | 1800 | 0.0713 | 0.0087 | 2.09×10^-11^ | |
| 100% | 4 | 0.00141 | 3.14 | 1800 | 0.222 | 0.0472 | | 6.36×10^-11^ |

**Table S6** GITT data for Si@CA@CMC-2

| Lithiation | Constant | L/cm | π | τ | ΔE_t_/V | ΔE_s_/V | *D*_Li+_/cm^2^s^-1^ |
| --- | --- | --- | --- | --- | --- | --- | --- |
| 10% | 4 | 0.00136 | 3.14 | 1800 | 0.0527 | 0.0025 | 2.95×10^-12^ |
| 20% | 4 | 0.00136 | 3.14 | 1800 | 0.0437 | 0.0015 | 1.54×10^-12^ |
| 30% | 4 | 0.00136 | 3.14 | 1800 | 0.0384 | 0.0019 | 3.20×10^-12^ |
| 40% | 4 | 0.00136 | 3.14 | 1800 | 0.0341 | 0.0019 | 4.06×10^-12^ |
| 50% | 4 | 0.00136 | 3.14 | 1800 | 0.0301 | 0.0021 | 6.37×10^-12^ |
| 60% | 4 | 0.00136 | 3.14 | 1800 | 0.0279 | 0.0007 | 8.24×10^-13^ |
| 70% | 4 | 0.00136 | 3.14 | 1800 | 0.0267 | 0.0006 | 6.61×10^-13^ |
| 80% | 4 | 0.00136 | 3.14 | 1800 | 0.0289 | 0.0006 | 5.64×10^-13^ |
| 90% | 4 | 0.00136 | 3.14 | 1800 | 0.0335 | 0.0013 | 1.97×10^-12^ |
| 100% | 4 | 0.00136 | 3.14 | 1800 | 0.0316 | 0.0037 | 1.79×10^-11^ |
| Delithiation | Constant | L/cm | π | τ | ΔE_t_/V | ΔE_s_/V | *D*_Li+_/cm^2^s^-1^ |
| 10% | 4 | 0.00136 | 3.14 | 1800 | 0.039 | 0.0059 | 3.00×10^-11^ |
| 20% | 4 | 0.00136 | 3.14 | 1800 | 0.041 | 0.0053 | 2.19×10^-11^ |
| 30% | 4 | 0.00136 | 3.14 | 1800 | 0.044 | 0.0062 | 2.60×10^-11^ |
| 40% | 4 | 0.00136 | 3.14 | 1800 | 0.049 | 0.0075 | 3.07×10^-11^ |
| 50% | 4 | 0.00136 | 3.14 | 1800 | 0.0499 | 0.0043 | 9.72×10^-12^ |
| 60% | 4 | 0.00136 | 3.14 | 1800 | 0.0453 | 0.0015 | 1.44×10^-12^ |
| 70% | 4 | 0.00136 | 3.14 | 1800 | 0.0502 | 0.0018 | 1.68×10^-12^ |
| 80% | 4 | 0.00136 | 3.14 | 1800 | 0.0607 | 0.0025 | 2.22×10^-12^ |
| 90% | 4 | 0.00136 | 3.14 | 1800 | 0.0883 | 0.0071 | 8.46×10^-12^ |
| 100% | 4 | 0.00136 | 3.14 | 1800 | 0.173 | 0.0152 | 1.01×10^-11^ |

**Table S7** GITT data for Si@CA@CMC-3

| Lithiation | Constant | L/cm | π | τ | ΔE_t_/V | ΔE_s_/V | *D*_Li+_/cm^2^s^-1^ |
| --- | --- | --- | --- | --- | --- | --- | --- |
| 10% | 4 | 0.00134 | 3.14 | 1800 | 0.0496 | 0.0031 | 4.96×10^-12^ |
| 20% | 4 | 0.00134 | 3.14 | 1800 | 0.0483 | 0.0028 | 4.27×10^-12^ |
| 30% | 4 | 0.00134 | 3.14 | 1800 | 0.0459 | 0.0022 | 2.92×10^-12^ |
| 40% | 4 | 0.00134 | 3.14 | 1800 | 0.0450 | 0.0031 | 6.03×10^-12^ |
| 50% | 4 | 0.00134 | 3.14 | 1800 | 0.0415 | 0.0038 | 1.07×10^-11^ |
| 60% | 4 | 0.00134 | 3.14 | 1800 | 0.0369 | 0.0028 | 7.32×10^-12^ |
| 70% | 4 | 0.00134 | 3.14 | 1800 | 0.0341 | 0.0016 | 2.80×10^-12^ |
| 80% | 4 | 0.00134 | 3.14 | 1800 | 0.0348 | 0.0012 | 1.51×10^-12^ |
| 90% | 4 | 0.00134 | 3.14 | 1800 | 0.0422 | 0.0018 | 2.31×10^-12^ |
| 100% | 4 | 0.00134 | 3.14 | 1800 | 0.0486 | 0.0031 | 5.17×10^-12^ |
| Delithiation | Constant | L/cm | π | τ | ΔE_t_/V | ΔE_s_/V | *D*_Li+_/cm^2^s^-1^ |
| 10% | 4 | 0.00134 | 3.14 | 1800 | 0.0326 | 0.0038 | 1.73×10^-11^ |
| 20% | 4 | 0.00134 | 3.14 | 1800 | 0.0362 | 0.004 | 1.55×10^-11^ |
| 30% | 4 | 0.00134 | 3.14 | 1800 | 0.035 | 0.0012 | 1.49×10^-12^ |
| 40% | 4 | 0.00134 | 3.14 | 1800 | 0.0328 | 0.0007 | 5.79×10^-13^ |
| 50% | 4 | 0.00134 | 3.14 | 1800 | 0.0325 | 0.0006 | 4.33×10^-13^ |
| 60% | 4 | 0.00134 | 3.14 | 1800 | 0.0359 | 0.0003 | 8.87×10^-14^ |
| 70% | 4 | 0.00134 | 3.14 | 1800 | 0.0412 | 0.0012 | 1.08×10^-12^ |
| 80% | 4 | 0.00134 | 3.14 | 1800 | 0.0512 | 0.0024 | 2.79×10^-12^ |
| 90% | 4 | 0.00134 | 3.14 | 1800 | 0.067 | 0.0037 | 3.88×10^-12^ |
| 100% | 4 | 0.00134 | 3.14 | 1800 | 0.2523 | 0.0233 | 1.08×10^-11^ |


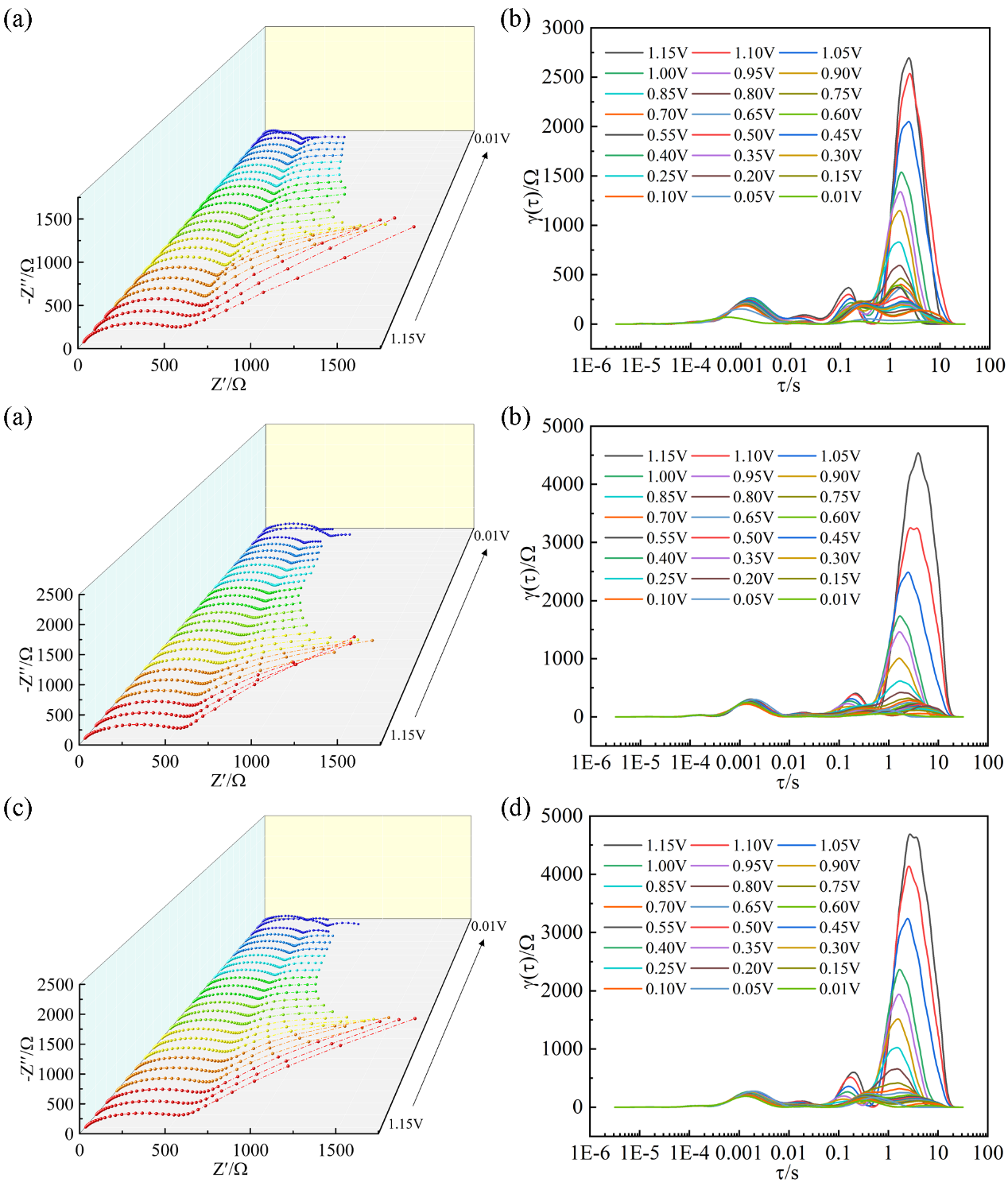


**Figure S6** *In-situ* EIS Nyquist plots and the distribution of relaxation time (DRT) related function γ(τ) at different voltage in the first discharge process of (a)-(b) Si@CA@CMC-2; (c)-(d) Si@CA@CMC-3


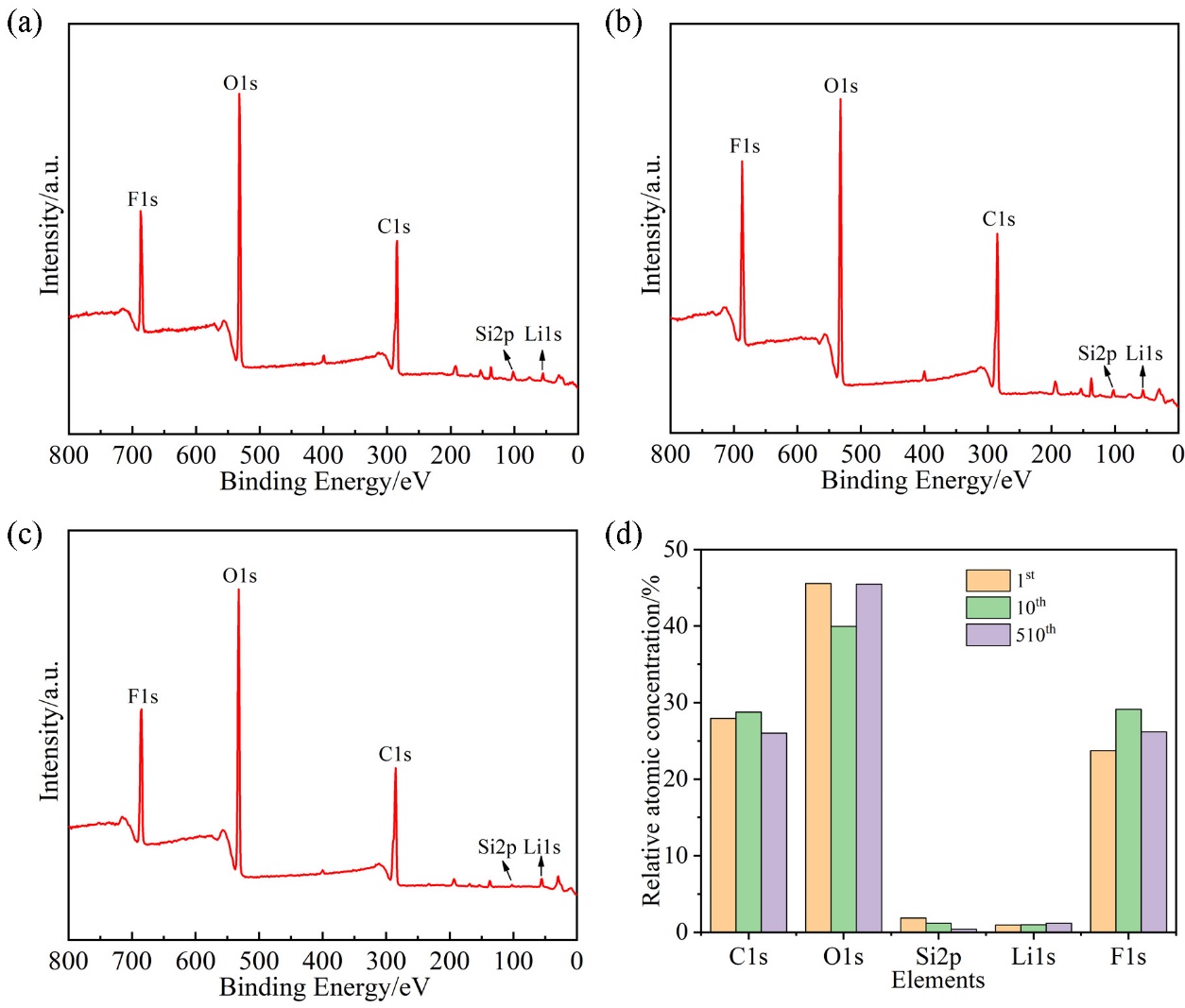


**Figure S7** (a)-(c) XPS full spectrum and (d) elements contents histogram calculated from XPS of Si@CA@CMC-1 after 1, 10, 510 cycles (disassembled at charge state (1V))

**Table S8** Statistics of the atomic contents in XPS of Si@CA@CMC-1 (different cycles)

| Cycles | C 1s | O 1s | Si 2p | Li 1s | F 1s |
| --- | --- | --- | --- | --- | --- |
| 1 | 27.93 | 45.55 | 1.85 | 0.95 | 23.72 |
| 10 | 28.76 | 39.95 | 1.18 | 0.98 | 29.13 |
| 510 | 26.01 | 45.47 | 0.42 | 1.19 | 26.91 |

**Table S9** Statistics of the atomic contents in XPS of Si@CA@CMC-1 (after 1/10/510 cycles)

|  | | 1st | 10th | 510th |
| --- | --- | --- | --- | --- |
| C 1s | C-C | 64.04 | 60.08 | 49.72 |
|  | C-O | 16.68 | 21.22 | 26.36 |
|  | C=O | 16.85 | 15.78 | 11.55 |
|  | RO-CO_2_Li | 2.43 | 2.92 | 12.37 |
| O 1s | -COOLi | 95.05 | 97.21 | 98.46 |
|  | Si-O | 4.95 | 2.79 | 1.54 |
| Si 2p | Si-Si | 20.78 | 6.78 | 10.90 |
|  | Si-O | 79.22 | 93.22 | 89.10 |
| Li 1s | Li-F | 3.86 | 6.00 | 6.18 |
|  | Li-O | 91.75 | 88.42 | 89.23 |
|  | Li-C | 4.39 | 5.58 | 4.59 |
| F 1s | Li-F | 25.12 | 28.02 | 61.20 |
|  | Li_x_PF_y_/Li_x_PO_y_F_z_ | 74.88 | 71.98 | 38.80 |


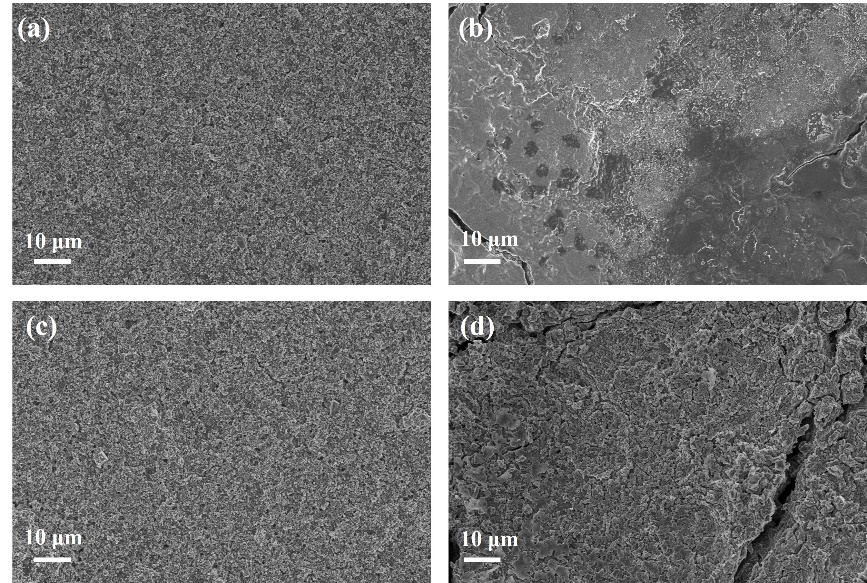


**Figure S8** SEM images of (a) Si@CA@CMC-2 electrode (fresh); (b) Si@CA@CMC-2 electrode (5th cycle); (c) Si@CA@CMC-3 electrode (fresh); (d) Si@CA@CMC-3 electrode (5th cycle) (at charge state (1V)).


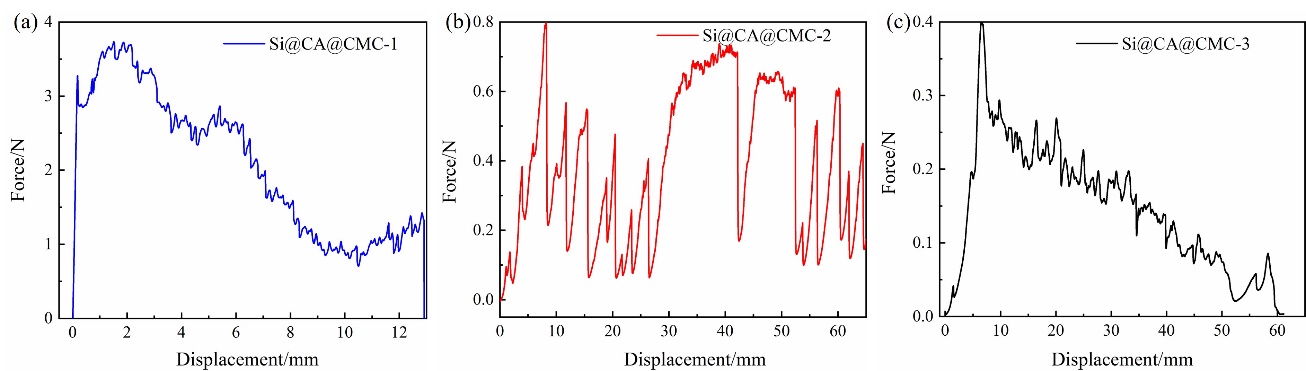


**Figure S9** Peeling test for fresh CA@CMC-X electrode (X=1, 2, 3)


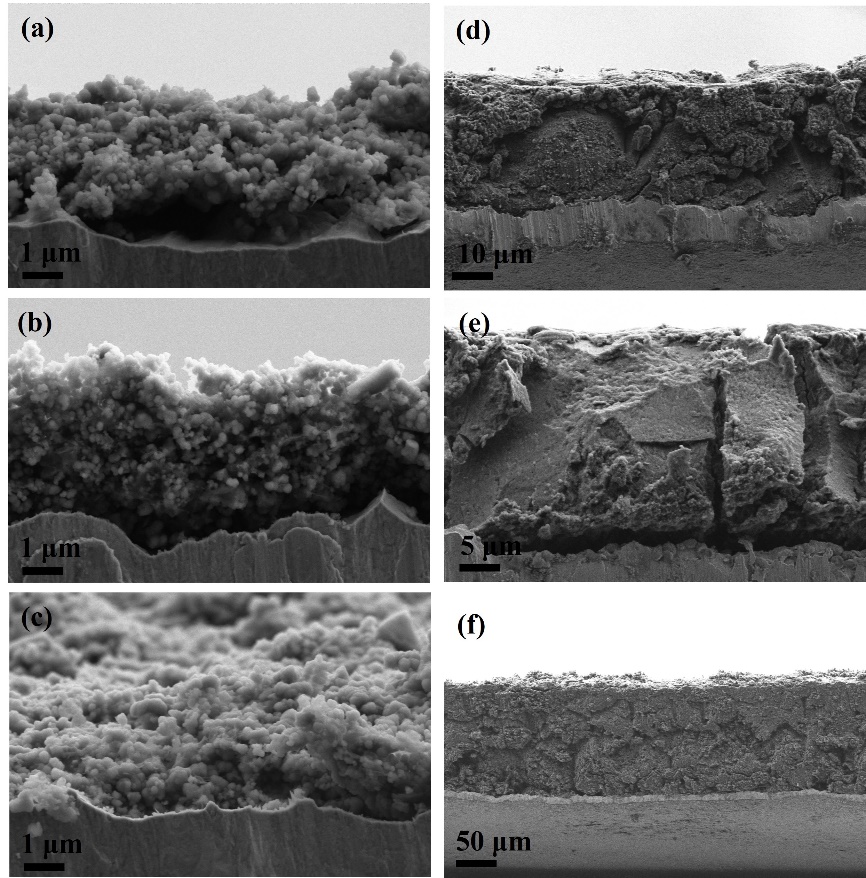


**Figure S10** Cross-section SEM images for CA@CMC-X (X=1, 2, 3): (a)-(c) fresh; (d)-(f) after 5 cycles (disassembled at charge state (1V))





**Figure S11** Cycling performance for pure CMC (0.1 A/g)





**Figure S12** Nyquist plots of pure CMC as binder (testing at open circuit potential)


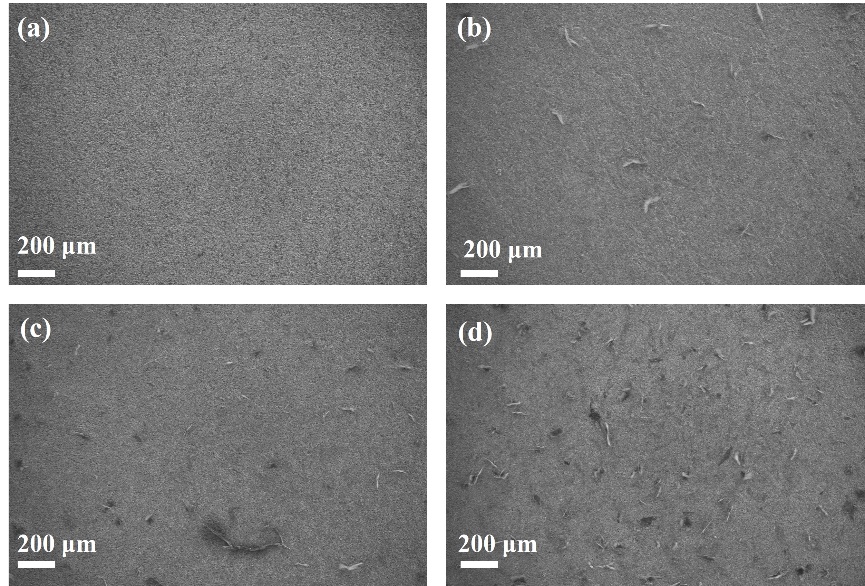


**Figure S13** SEM images of Si@CMC electrode material without further treatment: (a) fresh; (b) 1st cycle; (c) 5th cycles; (d) 10th cycles (at charge state (1V))


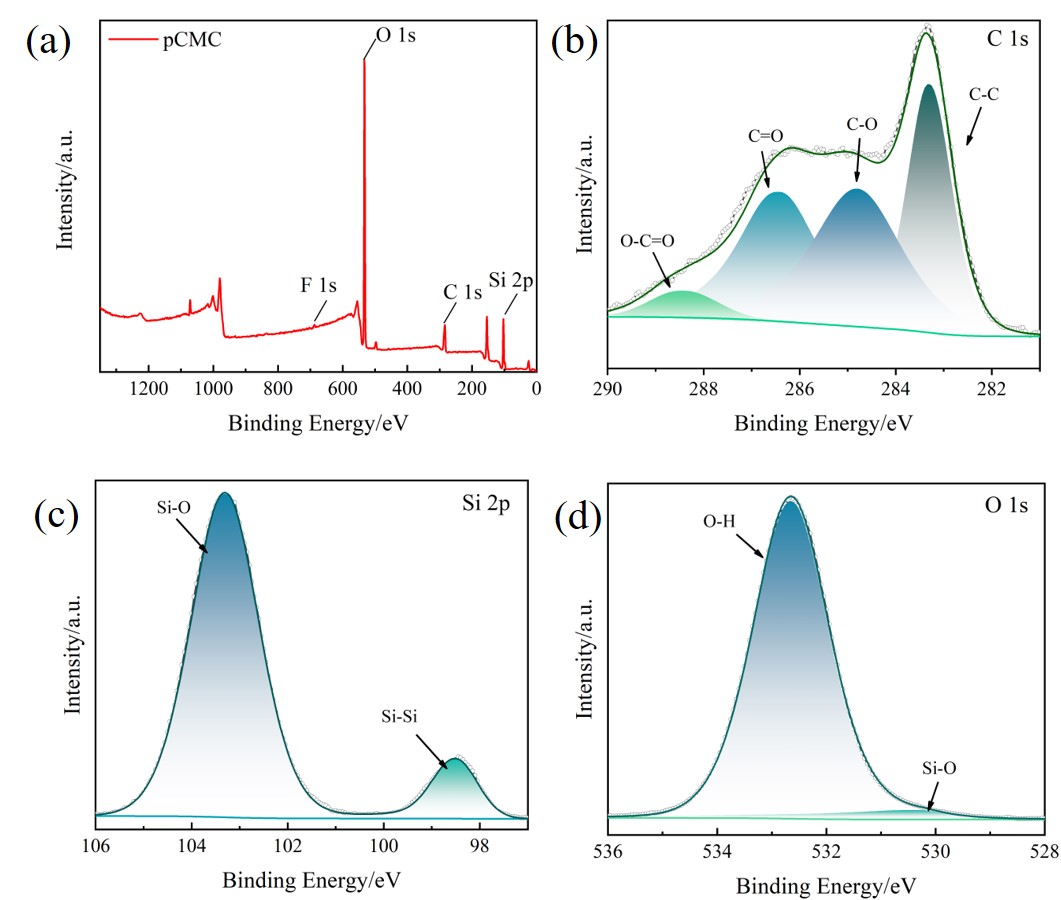


**Figure S14** XPS of pure CMC as binder (fresh)


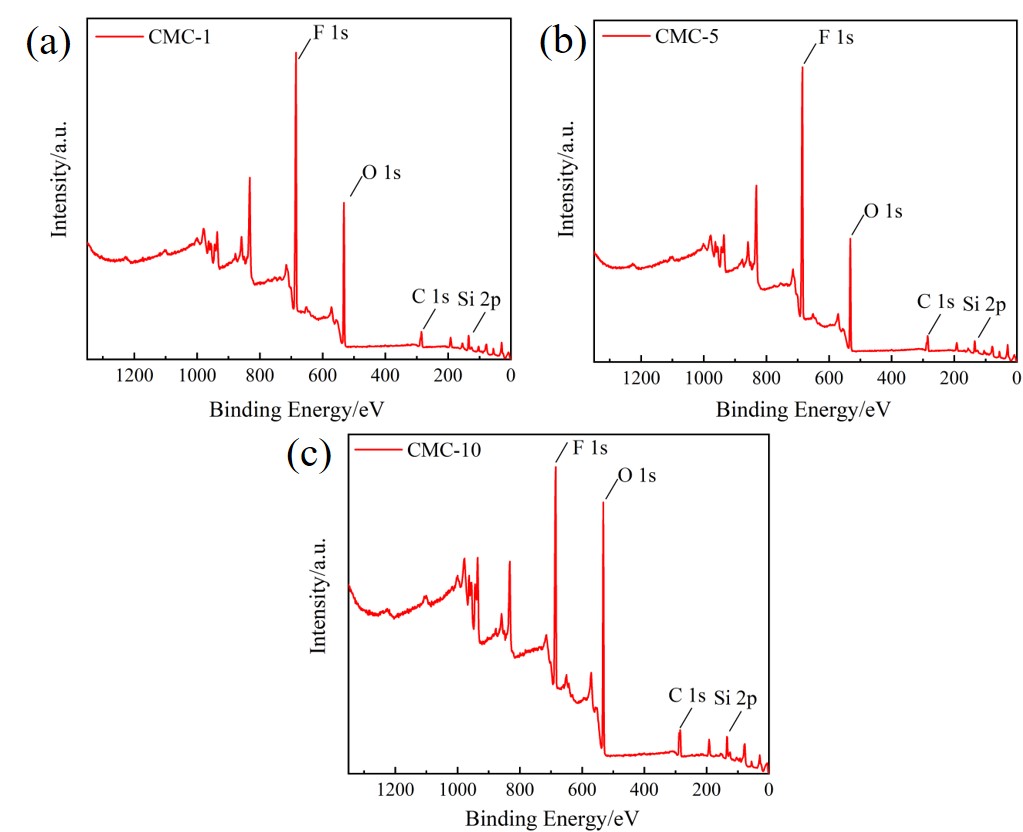


**Figure S15** (a)-(c) XPS full spectrum of pure CMC as binder after 1, 5, 10 cycles (disassembled at charge state (1V))


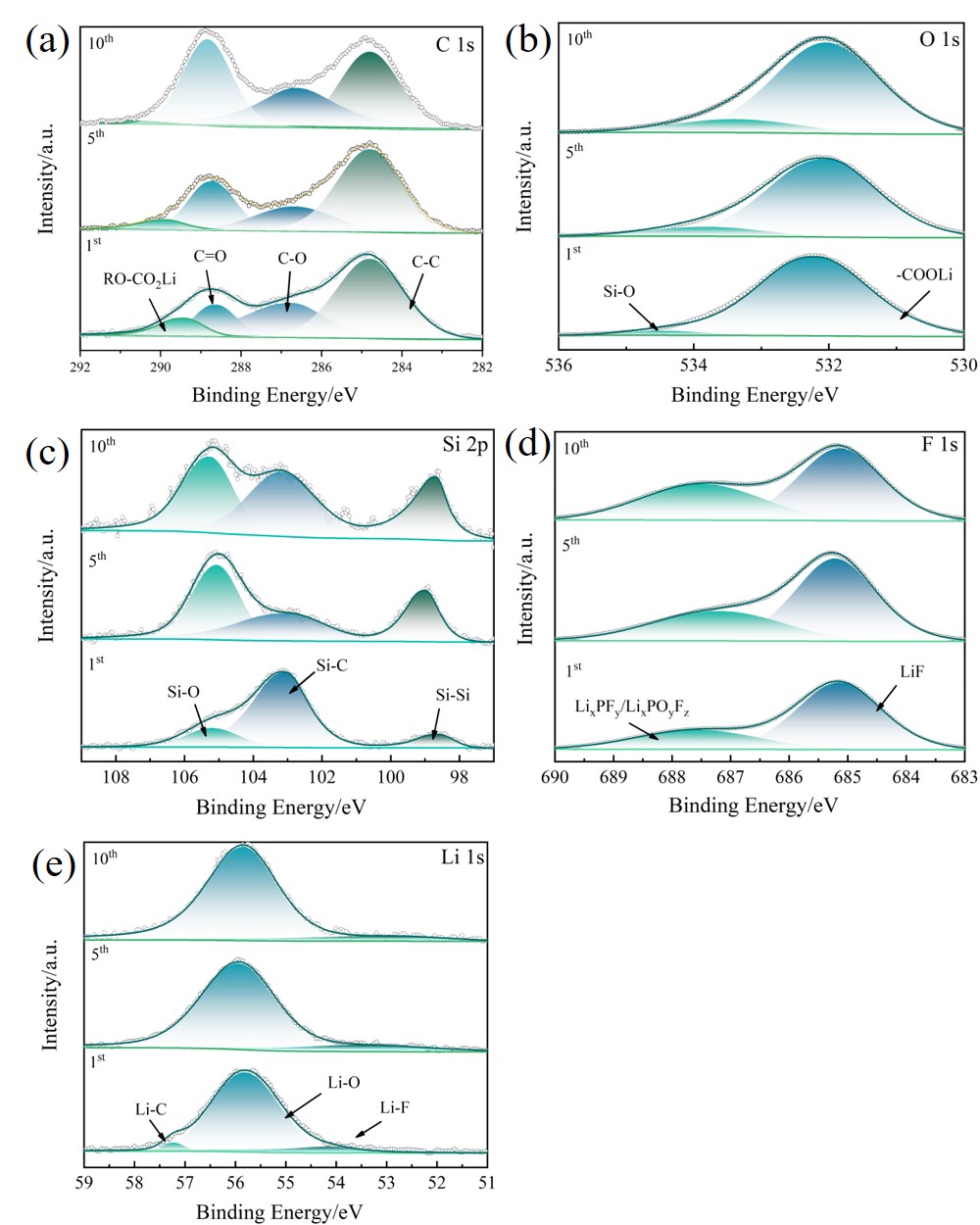


**Figure S16** XPS of pure CMC as binder after 1,5, 10 cycles (disassembled at charge state (1V)): (a) C 1s fine spectrum; (b) O 1s fine spectrum; (c) Si 2p fine spectrum; (d) F 1s fine spectrum; (e) Li 1s fine spectrum.
